# Resting Brain Perfusion and Selected Vascular Risk Factors in Healthy Elderly Subjects

**DOI:** 10.1371/journal.pone.0097363

**Published:** 2014-05-19

**Authors:** Otto M. Henriksen, Lars T. Jensen, Katja Krabbe, Per Guldberg, Tom Teerlink, Egill Rostrup

**Affiliations:** 1 Functional Imaging Unit, Section of Clinical Physiology and Nuclear Medicine, Department of Diagnostics, Glostrup Hospital, Copenhagen University Hospital, Glostrup, Denmark; 2 Department of Clinical Physiology, Nuclear Medicine and PET, Rigshospitalet, Copenhagen University Hospital, Copenhagen, Denmark; 3 Center for Healthy Aging, Faculty of Health and Medical Sciences, University of Copenhagen, Copenhagen, Denmark; 4 Department Clinical Physiology and Nuclear Medicine, Herlev Hospital, Copenhagen University Hospital, Herlev, Denmark; 5 Section of Radiology, Department of Diagnostics, Glostrup Hospital, Copenhagen University Hospital, Glostrup, Denmark; 6 Institute of Cancer Biology, Danish Cancer Society, Copenhagen, Denmark; 7 Metabolic Laboratory, Department of Clinical Chemistry, VU University Medical Center, Amsterdam, the Netherlands; University Medical Center Rotterdam, Netherlands

## Abstract

**Background and Purpose:**

Both cerebral hypoperfusion and vascular risk factors have been implicated in early aging of the brain and the development of neurodegenerative disease. However, the current knowledge of the importance of cardiovascular health on resting brain perfusion is limited. The aim of the present study was to elucidate the relation between brain perfusion variability and risk factors of endothelial dysfunction and atherosclerosis in healthy aged subjects.

**Methods:**

Thirty-eight healthy subjects aged 50–75 years old were included. Mean global brain perfusion was measured using magnetic resonance phase contrast mapping and regional brain perfusion by use of arterial spin labeling.

**Results:**

Mean global brain perfusion was inversely correlated with caffeine and hematocrit, and positively with end-tidal P_CO2_. Furthermore, the mean global brain perfusion was inversely correlated with circulating homocysteine, but not with asymmetric dimethylarginine, dyslipidemia or the carotid intima-media thickness. The relative regional brain perfusion was associated with circulating homocysteine, with a relative parietal hypoperfusion and a frontal hyperperfusion. No effect on regional brain perfusion was observed for any of the other risk factors. A multiple regression model including homocysteine, caffeine, hematocrit and end-tidal P_CO2_, explained nearly half of the observed variability.

**Conclusion:**

Both intrinsic and extrinsic factors influenced global cerebral perfusion variation between subjects. Further, the results suggest that the inverse relation between homocysteine and brain perfusion is owing to other mechanisms, than reflected by asymmetric dimethylarginine, and that homocysteine may be a marker of cerebral perfusion in aging brains.

## Introduction

Mounting evidence has suggested an association of vascular and brain health. Alzheimer's disease too shares many risk factors with cerebrovascular disease, an observation that has led to the suggestion that cerebrovascular dysfunction and hypoperfusion may play a role in the pathogenesis of neurodegenerative disorders.[1] Recent studies have shown that structural signs of brain aging are also associated with both lowered resting brain perfusion [2] and with general vascular risk factors. [3]

In healthy subjects, large between-subject variability of brain perfusion has been demonstrated, but the knowledge of the biological sources and the possible long-term consequences of this variability is limited.[4] Very recent studies have reported an inverse correlation between cardiovascular risk and measures of cerebral perfusion.[5], [6]

In addition to traditional vascular risk factors, also risk factors of arterial aging and endothelial dysfunction have been associated with accelerated structural and functional brain aging. [3] Among several humoral factors associated with endothelial dysfunction, homocysteine is a well-established risk factor for stroke and dementia and has also been associated with structural signs of brain aging. [7]–[9] The effects of homocysteine and of other risk factors have been suggested to be mediated by the endogenous nitric oxide (NO) synthase inhibitor asymmetric dimethylarginine (ADMA), which has also been demonstrated as an independent risk factor for vascular disease [10] and structural signs of brain aging.[11]

Increased carotid intima-media thickness (IMT), a measure of subclinical atherosclerosis, is also associated with impaired endothelial function and has been reported to correlate with structural brain aging, cognitive decline [3], [12] and with regional brain perfusion changes.[13] Finally, both prior and very recent studies have shown that severe hyperlipidemia may influence cerebrovascular tone by mechanisms unrelated to the pro-atherogenic properties of hyperlipidemia.[14], [15]

Although the above factors are known to influence both vascular function and brain health, their influences on brain perfusion in healthy subjects have not been established. The aim of this study was therefore to investigate the effect of natural variation of these factors on global and regional resting brain perfusion in a cross-sectional population based sample of elderly, healthy subjects. Further, the study should test the hypothesis that risk factors of endothelial dysfunction and atherosclerosis associated with increased risk of degenerative brain disease are also associated with decreased perfusion. Adjusting also for the effects of factors known to influence perfusion, such as hematocrit, carbon dioxide and caffeine, we expect that a significant fraction of the spontaneous variability in resting brain perfusion can be explained by a comprehensive selection of such measurable factors. Overall, the study showed that about half of the variability in brain perfusion could be explained by the factors investigated. Furthermore, it was found that homocysteine in particular was associated with changes in brain perfusion.

## Methods

### Subjects

Thirty-eight non-smoking subjects 50–75 years of age and in self-reported good health were recruited by newspaper advertisement. Subjects with a history of significant neurological or vascular disease or diabetes, use of neuro- or vasoactive medication or oral contraceptives or hormone replacement therapy were excluded from the study. Data relating brain blood flow to cardiac MRI measurements obtained in the same group of subjects have recently been published.[16]

### Ethics

The study was approved by the regional ethics committee (Ethics Committee of The Capital Region) following the standards of The National Committee on Health Research Ethics. The experiments were conducted in accordance with the Helsinki Declaration and all subjects gave written informed consent.

### Study design

The study was designed as a cross-sectional study with all experimental data for each subject obtained within a single session. On the day of the magnetic resonance imaging (MRI) experiments, the subjects were allowed to both eat and to drink coffee and tea as usual.

All subjects underwent an examination on a separate day prior to the experiment that included fasting blood sampling, ECG, blood pressure, height, weight, carotid Doppler ultrasound including measurement of intima-media thickness and Mini Mental State Examination (MMSE). Subjects with >50% internal carotid artery (ICA) stenosis, blood pressure >160/90 mmHg or fasting plasma cholesterol >7.5 mmol/l were excluded.

### MRI experiments

All MRI measurements were performed on a 3.0 T Philips Intera Achieva (Philips Medical Systems, Best, the Netherlands) using a 32 element phased array receive head coil and multitransmit parallel RF transmission for brain scans.

A high resolution structural scan used for tissue segmentation and calculation of brain volume was obtained using a 3D T_1_ weighted gradient echo sequence (repetition time (TR)  = 10 ms, echo time (TE)  = 5 ms, flip angle 8°, matrix 240×200, voxel size 1×1×1 mm, sensitivity encoding (SENSE) factor  = 2). To assess brain pathology, standard sagittal and axial T_2_ weighted scans were acquired in addition to a fluid attenuated inversion recovery (FLAIR) sequence (TR = 11 s, TE = 125 ms, TI = 2.8 s and resolution 0.7×0.8×4 mm).

Total brain blood flow was measured by MRI phase contrast mapping (PCM). Using PCM, volume flow in basilar and the internal carotid arteries (ICAs) can be measured accurately. Normalizing total flow to brain size, mean global brain perfusion can be calculated.[2] PCM measurements were obtained with a matrix of 320×320 (TR = 12 ms, TE = 7 ms, flip angle 10°, voxel size 0.75×0.75×8mm) with a V_enc_ of 100 cm/s. The sequence was ECG gated (retrospective gating, 20 frames/cycle).

Regional brain perfusion maps were obtained using the Quasar sequence, which is a multi slice, multiple inversion time (TI) pulsed arterial spin labeling (ASL) scheme.[17] General scan parameters were: matrix 80×80, voxel size 3×3×6 mm, gap 1.5 mm, TR/TE/ΔTI/TI1  = 4000/22/300/40 ms, flip angle 35°/11.7°, SENSE 2.5, 84 averages (48@Venc = 4 cm/s, 24@Venc = ∞, 12 low flip angle). Seven transaxial slices were acquired parallel to the lower edges of the corpus callosum.

Blood pressure was monitored with a Veris 8600 Vital Signs Monitor (Medrad, Indianola, PA). Expiratory gas was sampled from a mouthpiece and end-tidal P_CO2_ (P_ET_CO_2_ ) was monitored with a Biopac MP150 system (Biopac Systems, Inc., Goleta, CA).

### Post-processing

All MRI data were converted into NIfTI format and analyzed using in-house software for Matlab version 7.9 (The MathWorks Inc., Natick, MA) except when indicated otherwise.

#### Phase contrast mapping

PCM measurements were processed as previously described.[4] Initially an automatic pixel wise phase correction procedure was applied. Then regions of interest for both ICAs and the basilar artery were assigned manually by one investigator and flow was calculated by multiplying mean velocity with vessel area and integrating over time. Brain perfusion is calculated as total brain flow divided by brain volume and reported in mL/100 g/min assuming a tissue density of 1 g/mL.

#### Arterial spin labeling

ASL data was analyzed using the FSL QUASIL tool applying model-based quantitation.[18] This procedure produces maps of perfusion as well as estimated tissue relaxation rate R1. The perfusion maps were spatially normalized to the MNI standard brain provided by FSL (FMRIB Software Library, www.fmrib.ox.ac.uk/fsl) using an intermediate step in which the R1-images were co registered to the individual high-resolution anatomical scan. In order to obtain relative perfusion maps, perfusion values were normalized to a mean brain perfusion value of 50 mL/100 g/min.

#### Structural scan

The 3D T_1_ weighted scan was segmented using the FSL BET and FAST tools. The resulting cerebrospinal fluid (CSF), gray matter and white matter probability maps were used to calculate the total brain tissue volume (V_tot_), CSF volume (V_csf_) and brain parenchymal fraction (BPF) calculated as 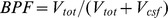



Structural scans were reviewed by an experienced neuroradiologist for pathology and severity of white matter lesions using a modified Fazekas rating scale.[19]

### Blood samples

Venous blood samples were collected on the day of inclusion and the day of the MRI experiment immediately before commencing the scanning. Blood samples were centrifuged and frozen within 2 hours of sampling and stored at −20°C until analysis.

Fasting blood samples from the day of inclusion were analyzed for plasma lipids, glucose, creatinine, and APOE genotype. The ratio of low density cholesterol to high density lipoprotein cholesterol (LDL:HDL ratio) was calculated as a measure of dyslipidemia.

Venous blood samples were drawn immediately before the MRI scan were analyzed for hematocrit and for plasma concentrations of ADMA, L-arginine, homocysteine and caffeine.

#### Blood sample analysis

Total plasma homocysteine was determined using an automated latex enhanced immunoassay (Hemosil Homocysteine, Instrumentation Laboratory, Lexington, MA). Intra- and inter-assay CV were 2.9% and 6% respectively.

Asymmetric dimethylarginine (ADMA) and L-arginine were determined by high-performance liquid chromatography with fluorescence detection as previously described using modified chromatographic separation conditions.[20], [21] Intra- and inter-assay CV were 1.3% and 2.5%, respectively for arginine, and 1.4% and 2.9%, respectively for ADMA. The Arg:ADMA ratio was calculated because NO production is determined by both substrate availability (arginine) and the presence of inhibitor (ADMA), which may be adequately reflected by this ratio.

In order to characterize the study population genetically, APOE genotype (rs429358 and rs7412 variants) was determined by pyrosequencing using the PyroMark Q24 system (Qiagen, Hilden, Germany) and the following primers: GGAACTGGAGGAACAACTGACC (forward), [Btn]-TACACTGCCAGGCGCTTCT (reverse) and GCGGACATGGAGGAC (sequencing) for rs429358, and AGCTGCGTAAGCGGCTCCT (forward), [Btn]-CCCCGGCCTGGTACACTG (reverse) and CGATGACCTGCAGAA (sequencing) for rs7412.

Analysis of plasma caffeine was performed using a high-performance liquid chromatography/tandem mass spectrometry method originally developed and validated for analysis of urine samples adapted to analysis of plasma as previously described.[22] Intra- and inter-assay CV were both <7%.

### Intima-media thickness

All ultrasound examinations were performed and analysed by the same investigator using a Philips iU22, Ultrasound system (Philips Medical Systems, Best, The Netherlands) equipped with a 3–9 MHz transducer. Long axis B-mode images of the distal part of both common carotid arteries were acquired in three views (anterior, lateral and posterior). IMT was measured at the far wall using dedicated edge detection software (QLAB, Philips Medical Systems, Best, the Netherlands).

### Cardiovascular risk

For each subject sex-specific Framingham 10 year cardiovascular event risk was estimated from subject's age, fasting lipids and blood pressure (measured during the MRI session).[23]

### Statistical analysis

Multiple regression analysis was applied for analysis of mean global brain perfusion values. Effects of covariates on regional perfusion were analyzed by applying a general linear model to the spatially normalized perfusion maps. The analysis was performed using in-house software written in Matlab in order to take into account the slightly non-overlapping brain coverage between subjects. Thus, data usage was optimized by including voxels in standard space, even if not all subjects contributed data to that point. Data was thresholded for visualization using a criterion of p<0.001 (uncorrected), cluster size 20 voxels, including only voxels to which at least 10 subjects contributed.

For ordinal data (i.e. WML grade), ordinal logistic regression was applied. Group differences were analyzed using Mann-Whitney test for continuous variables. All statistical analysis was performed using STATA 12 SE (StataCorp, College Station, CA).

## Results

Study population characteristics are presented in Table 1.

**Table 1:** Subject characteristics.

	Median	Range
Sex (n = male/female)	19/19	
Age (years)	64	50–75
Body mass index (kg/m^2^)	25.4	18.3–34.3
MMSE score	29	24–30
Total cholesterol (mmol/L)	5.6	3.9–7.5
LDL:HDL ratio	2.1	0.7–5.7
Intima-media thickness (mm)	0.69	0.5–0.97
Number of APOE4 alleles (0/1/2)	22/13/3	
10 year CV risk score (%)	12.6	3.3–31.3
Brain volume (mL)	1086	902–1528
Brain parenchymal fraction	0.738	0.701–0.784
Fazekas WML grade (0/1/2/3)	14/18/5/1	
Hematocrit (%)	44	38–55
P_ET_CO_2_ (kPa)	4.42	2.83–5.78
Caffeine (μmol/L)	17.8	0.01–74.2
ADMA (μmol/L)	0.43	0.33–0.53
Arg:ADMA ratio	146	47–206
Homocysteine (μmol/L)	9.0	5.7–13.7
Mean arterial blood pressure (mmHg)	99	79–121
Total flow (mL/min)	500	307–798
Brain perfusion (mL/100 g/min)	46.8	24.6–67.2

Abbreviations: MMSE  =  mini mental state examination, LDL  =  low density lipoprotein, HDL  =  high density lipoprotein, CV =  cardiovascular WML  =  white matter lesion, ADMA  =  asymmetric dimethylarginine, Arg =  L-arginine.

### Global brain perfusion

**Figure 1:**
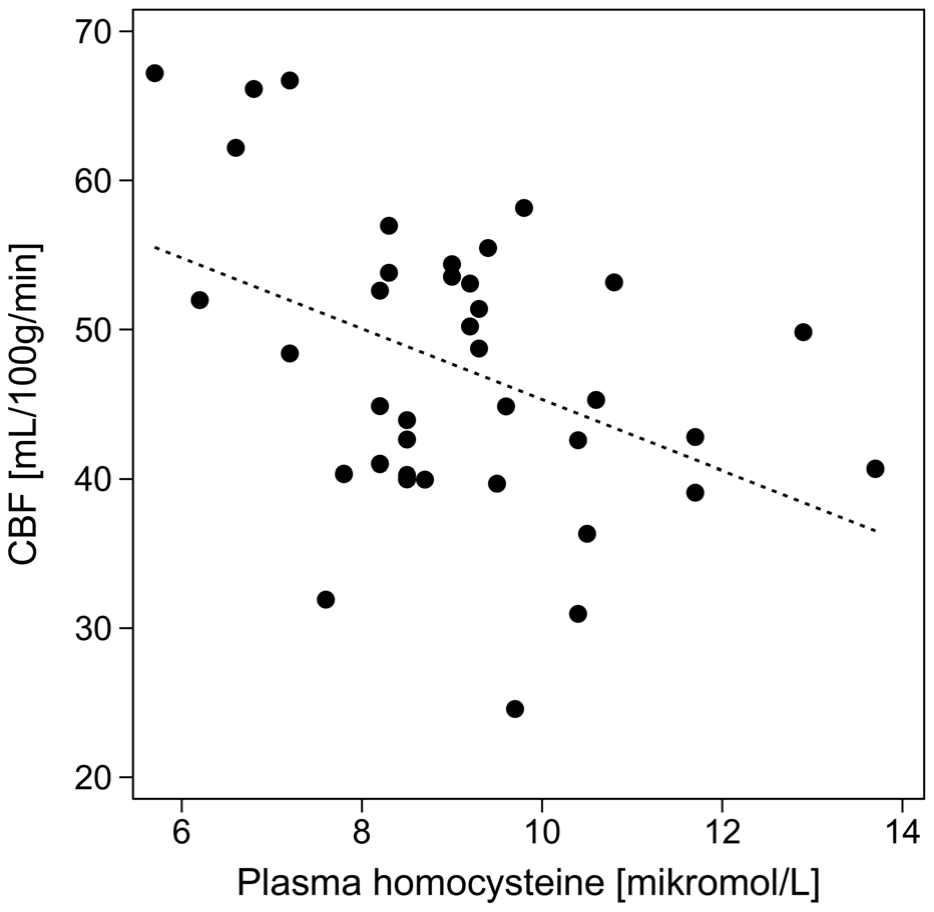
The relationship between homocysteine and global brain perfusion. Dashed line shows line of regression (r^2^ = 0.17, p = 0.009).

The effects of the various covariates on brain perfusion in univariate and multiple regression analysis (adjusted for age and sex) are presented in Table 2. Mean brain perfusion was inversely correlated with homocysteine (Figure 1). The effect remained significant after adjustment for age and sex (Table 2), and also when adjusted for hematocrit too (−1.96 [95% CI: −3.79, −0.14] mL/100 g/min per µmol/L, p = 0.036).

**Table 2:** Effects of covariates on brain perfusion.

	Unadjusted		Adjusted for age and sex		
	Coef.	95% conf. interval	Coef.	95% conf. interval	Partial r^2^
Age (year)	−.0257	−0.768, 0.254	−0.243	−0.740, 0.255	0.027
Male sex	−5.55	−11.8, 0.72	−5.44	−11.72, 0.83	0.074
Hematocrit (%)	−1.06†	−0.99, −0.13	−1.01* †	−1.95, −0.08	0.121
P_ET_CO_2_ (kPa)	7.16†	2.33, 10.06	6.88^††^	1.97, 11.79	0.208
Caffeine (µmol/L)	−0.212†	−0.420, −0.003	−0.295^††^	−0.494, −0.097	0.212
CVD risk (% point)	−0.24	−0.67, 0.20	0.30	−0.41, 1.03	0.021
LDL:HDL ratio	0.99	−2.25, 4.22	1.93	−1.27, 5.15	0.042
Intima-media thickness (mm)	−15.1	−49.7, 19.5	4.25	−36.8, 45.3	0.001
Homocysteine (µmol/L)	−2.37^††^	−4.13, −0.32	−2.00†	−3.86, −0.13	0.123
ADMA (µmol/L)	10.4	−50.9, 71.8	16.7	−43.2, 76.6	0.009
Arg:ADMA (per 100)	0.08	−9.58, 9.59	−2.22	−11.99, 7.54	0.006

Regression coefficients refer to change in brain perfusion in mL/100 g/min per unit increase of covariate.

*Sex omitted due to co-linearity.

† p<0.05, ^††^ p<0.01 (p-values are not adjusted for multiple comparisons).

Abbreviations: LDL  =  low density lipoprotein, HDL  =  high density lipoprotein, ADMA  =  asymmetric dimethylarginine, Arg  =  L-arginine.

Although homocysteine in correlation analysis was positively correlated with both LDL:HDL ratio (r^2^ = 0.11, p = 0.041) and ADMA (r^2^ = 0.13, p = 0.025) and also tended to correlate negatively with Arg:ADMA ratio (r^2^ = 0.10, p = 0.056), neither of these were correlated with brain perfusion (Table 2).

IMT was positively correlated with both age (0.006 [95% CI: 0.001, 0.010] mm per year, p = 0.014) and male sex (0.080 [95% CI: 0.022, 0.138] mm, p = 0.008) in a multiple regression model including both covariates, and was also correlated inversely with Arg:ADMA ratio (r^2^ = 0.16, p = 0.015). No effect of IMT on brain perfusion was observed (Table 2), but including also a sex x IMT interaction in the analysis, a non-significant inverse correlation of IMT with mean global brain perfusion appeared in females only (−50.2 [95% CI: −110.4, 9.9] mL/100 g/min mL in females vs. 22.1 [95% CI: −22.6, 66.7] mL/100 g/min in males, p = 0.099 in females, and p = 0.058 for interaction).

Total brain flow was not different (501 vs. 500 mL/min) in men and women. However, brain perfusion tended to be higher in females than males (52.6 vs. 43.9 mL/100 g/min, p = 0.093) due to differences in brain volume, but no effect of age was observed (Table 2).

Brain perfusion was inversely correlated with hematocrit and caffeine, and positively with P_ET_CO_2_. (Table 2) Adjusting for hematocrit, no effect of sex on brain perfusion was observed. Table 3 shows the relative effect size of covariates in a multiple regression model including P_ET_CO_2_, hematocrit, caffeine and homocysteine. This model explained nearly half of brain perfusion variability (unadjusted r^2^ = 0.49). The effects of both homocysteine and of caffeine are larger than or equal to those of hematocrit and P_ET_CO_2_.

**Table 3:** Regression model using standardized regressors.

	Coef.	95% conf. interval	p-value	Partial r^2^
Hematocrit (%)	−2.71	−5.53, 0.11	0.059	0.142
Homocysteine (µmol/L)	−3.61	−6.59, −0.64	0.019	0.114
Caffeine (µmol/L)*	−2.58	−5.44, 0.29	0.076	0.101
P_ET_CO_2_ (kPa)	3.13	0.27, 5.99	0.033	0.170
Intercept (mL/100 g/min)†	47.4	44.8, 50.1	-	-

*Caffeine levels are not normally distributed, † at mean hematocrit and P_ET_CO_2_.

### Regional brain perfusion

**Figure 2:**
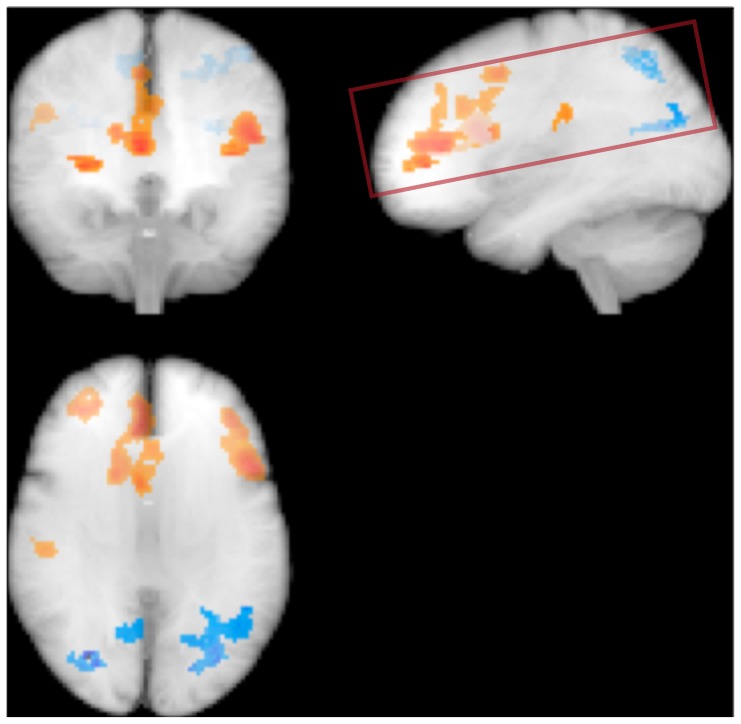
Effect of homocysteine on regional brain perfusion. Glass brain representation of voxels with relative regional brain perfusion increase (orange) or decrease (blue) in normalized brain perfusion maps. Rectangle shows the approximate volume covered by the ASL acquisition. Significance level p<0.001, effect adjusted for age and sex.

Analysis of regional brain perfusion maps showed that increasing homocysteine levels were associated with relative parietal hypoperfusion and frontal hyperperfusion. (Figure 2) Neither cardiovascular event risk, Arg:ADMA, LDL:HDL or IMT was associated with regional brain perfusion changes.

## Discussion

This study examines the association of risk factors of endothelial dysfunction and atherosclerosis with resting brain perfusion in elderly, healthy subjects. We found an inverse association of homocysteine with brain perfusion, whereas other vascular risk factors did not appear to be major determinants of between-subject brain perfusion variability. Additionally, the study confirms that there is a strong association between brain perfusion and factors such as hematocrit, P_CO2_ and caffeine. Previously, such associations have been described mainly in interventional studies, whereas the cross-sectional between-subject effects are less well documented.

This is the first report of direct association between homocysteine and brain perfusion in healthy subjects. As most studies of homocysteine lowering treatment have failed to demonstrate any beneficial effect on cardiovascular morbidity and mortality, it has been argued that homocysteine should rather be considered a risk marker than a risk factor of vascular disease.[24] However, long-term treatment has been reported to be effective in primary prevention of stroke [25] and to slow the rate of cerebral atrophy in patients with mild cognitive impairment [26] suggesting that the effects on the brain may differ from those on other organs.

It is not clear how homocysteine exerts its deleterious effects on the brain. Animal and human studies have shown that hyper-homocysteinemia may reduce bioavailability of NO and increases levels of reactive oxygen species [27], leading to endothelial dysfunction [28] and vascular remodeling [29], which in turn causes cerebrovascular resistance to increase.[30] To our knowledge, the present study is the first to show an association of homocysteine levels with brain perfusion, thus supporting cerebral hypoperfusion as a mediator of homocysteine associated cerebral pathology. One previous study involving demented subjects with cobalamin deficiency, reported that brain perfusion increased in patients who responded clinically to cobalamin treatment. Of notice, responders also had the higher homocysteine levels than non-responders.[31]

The regional brain perfusion pattern associated with increasing homocysteine shows a relative parietal hypoperfusion and frontal hyperperfusion. The parietal hypoperfusion bears some resemblance with the posterior hypometabolism associated with Alzheimer's disease and could indicate an early, subclinical manifestation of a neurodegenerative process. More conspicuous is the relative frontal hyperperfusion. Similarly, a recent study showed increased perfusion in parietal, frontal and temporal cortices of healthy APOE4 carriers [32] unlike in demented subjects where the APO4 allele is associated with hypoperfusion in the same regions. [33] This phenomenon may be explained as an early adaptive response to capillary flow inhomogeneity in order to maintain tissue oxygenation. [34]

From the present data the mechanism of action cannot be determined, but the findings do not support that homocysteine reduces brain perfusion by an ADMA induced decrease of NO availability. This is in line with findings of a recent study in patients with severe hyperhomocysteinemia due to cystathionine β-synthase deficiency, suggesting that the negative vascular effects of hyperhomocysteinemia have an ADMA-independent etiology.[35]

We did not observe any relation between ADMA or Arg:ADMA ratio with brain perfusion or on structural brain aging in our sample. However, in healthy subjects variability of ADMA is relatively small and unlike most previous studies showing an association of ADMA with vascular disease [10], [11], we did not include current smokers and subjects with known hypertension or diabetes. It is thus possible that an effect of ADMA on structural signs of brain aging and possibly also on brain perfusion could be detected in a larger sample also including subjects with a higher vascular risk burden.

No association of general cardiovascular event risk with neither regional nor global brain perfusion was detected. This is most likely due to a relatively small effect size, such that the effect can only be detected in very large samples.[6]

Recent studies reported an association of IMT with both mean global [6] and with regional brain perfusion changes, in particular in females.[13] We did not observe any effect of IMT on mean global or regional brain perfusion. Nor did we observe an effect of HDL:LDL ratio (or of LDL) on mean global or regional brain perfusion. This finding is in line with results from a large study showing no correlation of total brain flow with plasma LDL, total cholesterol or statin treatment. [36]

We observed a negative correlation of caffeine with brain perfusion thus confirming that spontaneous variation in caffeine levels can have large effects on resting brain perfusion and should be taken into account in studies involving measurement of brain perfusion.

The study also provides some insight into the biological sources of variation of brain perfusion measurements. The combined effects of sex, hematocrit and P_ET_CO_2_ may account for only approximately 15% of the spontaneous total between-subject variance.[37] The observation that homocysteine and caffeine may be equally important factors as hematocrit and P_ET_CO_2_, significantly adds to the understanding of the factors underlying the variability of brain perfusion. In the model presented in Table 3, the effects of hematocrit and caffeine were no longer significant at the 5% level. As these effects could not be attributed to co-linearity of the covariates, we included also these factors in the final model. The presented model does not imply causality and does not take into account the possible confounding effects of other factors of possible influence.

### Study limitations

Accuracy of PCM may be compromised by partial volume effects if in-plane resolution is too low relative vessel diameter (<4–5 pixels) or if the imaging plane is not perpendicular to the vessel.[38] In larger vessels like the ICAs these requirements are usually fulfilled with the imaging protocol used, but partial volume effects may cause inaccuracies of flow measurements in the basilar artery. However, the CBF reported values are in general within the expected range, and the observed correlations of total flow with brain volume and of CBF with gender, hematocrit and P_ET_CO_2_ further support the use of PCM for CBF measurements.

The Quasar ASL scheme does not allow whole brain coverage. In order to minimize the effect of flow velocity on labeling, the labeling slab included the circle of Willis. Consequently, the measurements slices did not cover the inferior parts of the brain including the temporal lobes.

Finally, the relatively small sample size warrants the findings to be confirmed in future studies.

### Perspectives

The association of homocysteine on cerebrovascular function should be investigated in future studies. In that context, it is intriguing why homocysteine and caffeine, which are both associated with reduced brain perfusion, appear to have opposing effects on cerebral health.[39] Future studies should address the effects of caffeine and homocysteine levels on brain perfusion in a stroke population.

## Conclusions

The present study adds to our understanding of the sources of brain perfusion variability by showing that homocysteine and brain perfusion co-vary in elderly healthy subjects. These findings have implications for the interpretation of the interplay between vascular risk factors, brain aging and cerebral perfusion.

From the present study, homocysteine may be considered be a marker of altered cerebral perfusion, but the small sample size warrants the findings to be investigated in larger studies, also including diseased subjects and with long term follow-up.
